# Complete Genome Sequence of Streptococcus pneumoniae Strain BVJ1JL, a Serotype 1 Carriage Isolate from Malawi

**DOI:** 10.1128/MRA.00715-21

**Published:** 2021-09-30

**Authors:** Modupeh Betts, Seth Jarvis, Aaron Jeffries, Andrea Gori, Chrispin Chaguza, Jacquline Msefula, Caroline M. Weight, Brenda Kwambana-Adams, Neil French, Todd D. Swarthout, Jeremy S. Brown, Robert S. Heyderman

**Affiliations:** a NIHR Global Health Research Unit on Mucosal Pathogens, Division of Infection and Immunity, University College London, London, United Kingdom; b UCL Genetics Institute, University College London, London, United Kingdom; c University of Exeter, Exeter, United Kingdom; d Wellcome Sanger Institute, Wellcome Genome Campus, Hinxton, United Kingdom; e Darwin College, University of Cambridge, Cambridge, United Kingdom; f Department of Clinical Infection, Microbiology and Immunology, Institute of Infection, Veterinary and Ecological Sciences, University of Liverpool, Liverpool, United Kingdom; g Malawi-Liverpool-Wellcome Trust Clinical Research Programme Blantyre, Blantyre, Malawi; h Department of Respiratory Medicine, Centre for Inflammation and Tissue Repair, University College London, London, United Kingdom; University of Rochester School of Medicine and Dentistry

## Abstract

Streptococcus pneumoniae is a leading cause of pneumonia, meningitis, and bacteremia. Serotype 1 is rarely carried but is commonly associated with invasive pneumococcal disease, and in the African “meningitis belt,” it is prone to cause cyclical epidemics. We report the complete genome sequence of S. pneumoniae serotype 1 strain BVJ1JL, isolated in Malawi.

## ANNOUNCEMENT

Streptococcus pneumoniae, a Gram-positive bacterium, is a leading cause of childhood mortality worldwide (1). At least 100 different capsular serotypes of S. pneumoniae have been described (2). Serotype 1 is among the most commonly isolated serotypes from blood or cerebrospinal fluid (CSF) (3, 4).

Strain BVJ1JL was isolated in 2015 from a nasopharyngeal swab (NPS) obtained from a 9-year-old child in Blantyre, Malawi (5). The study protocol was approved by the College of Medicine Research and Ethics Committee, University of Malawi (P.02/15/1677), and the Liverpool School of Tropical Medicine Research Ethics Committee, UK (14.056). The primary NPS, retrieved from storage in skim milk-tryptone-glucose-glycerol (STGG) medium, was plated onto Columbia blood agar (CBA) supplemented with 5% horse blood and incubated overnight at 37°C and 5% CO_2_. A single colony was then picked and purified on a fresh CBA plate. S. pneumoniae was confirmed by morphology, optochin test, and Gram stain. The capsular type was assessed using a serological latex agglutination test kit (ImmuLex Pneumotest; SSI Diagnostica) and confirmed genomically using PneumoCat (6). DNA was isolated from lawn plate cultures of frozen stocks incubated overnight at 37°C and 5% CO_2_ on CBA. The Qiagen Genomic-tip 500/G DNA kit was used to isolate DNA for PacBio sequencing, following the manufacturer’s protocol. Lysis buffer was supplemented with 30 mg/ml lysozyme (Sigma-Aldrich) and 50 units mutanolysin (Sigma-Aldrich). DNA was sheared using a g-TUBE device (Covaris) with a target length of 10 kb, and library preparation was performed according to the protocol “Preparing Multiplexed Microbial Libraries Using SMRTbell Express Template Prep Kit 2.0,” with the Barcoded Overhang Adapter Kit-8A (Pacific Biosciences) at the University of Exeter, UK. The pooled samples were purified and size selected to remove SMRTbell templates of >3 kb using AMPure PB beads (Pacific Biosciences). A 10-h capture using a 1M single-molecule real-time (SMRT) cell was performed on a PacBio Sequel instrument using Sequel v3 chemistry (7). In total, 295,697 reads were generated for BVJ1JL with a read *N*_50_ length of 5,084 bp. The reads were demultiplexed and downsampled using HGAP4 in the SMRTLink v8.0 portal. DNA for Illumina sequencing was isolated using the DNeasy blood and tissue kit (Qiagen) and quantified using Quant-iT PicoGreen double-stranded DNA (dsDNA) kits (Invitrogen) according to the manufacturer’s specifications. DNA was fragmented using an EpiSonic sonication system (EpiGentek), and libraries were constructed using the NEBNext Ultra DNA sample prep master mix kit (New England BioLabs [NEB]) with in-house adapters and barcode tags described at Oxford Genomics Centre, UK (8). The libraries were sequenced on an Illumina HiSeq 4000 instrument as 150-bp paired-end reads. The raw Illumina DNA reads (1,162,988 paired-end reads) were trimmed of low-quality ends and cleaned of adapters using Trimmomatic v0.32 (9). In all, 1,400,000 reads (700,000 paired-end reads) were used for the genome assembly. *De novo* assembly using the PacBio and Illumina reads was performed with the Unicycler v0.4.9b pipeline in bold mode (10), resulting in a single circular contig, as confirmed using Bandage v0.8.1 (11), with a final genome coverage of >500×. The generated assembly was quality assessed using QUAST v5.1.0rc1 (12), and automated annotation was performed using Prokka v1.14.6 (13). The genome assembly was 2,134,668 bp with a G+C content of 39.73%. Prokka v1.14.6 predicted 2,101 coding sequences, 2,238 genes, 12 rRNAs, 59 tRNAs, 59 noncoding RNAs, and 9 riboswitches. Default parameters were used for all software unless otherwise specified.

BVJ1JL belonged to sequence type 5012 (ST5012) and Global Pneumococcal Sequence Cluster 2 (GPSC2) lineage (14). ST5012 is a locus variant of the highly virulent ST217 (15, 16). Genetic analysis predicted susceptibility to penicillin (17) but resistance to chloramphenicol and tetracycline (*cat* and *tetM*, respectively). The macrolide resistance genes *mef* and *ermB* were not detected.

### Data availability.

The assembled complete genome sequence has been deposited in GenBank under accession number CP071871. The PacBio and Illumina raw reads are available in NCBI Sequence Read Archive (SRA) under accession numbers SRX10254117 and SRX10254116, respectively. The BioProject accession number is PRJNA695191, and the BioSample accession number is SAMN17602804.
