# Erratum for Betts et al., “Complete Genome Sequence of Streptococcus pneumoniae Strain BVJ1JL, a Serotype 1 Carriage Isolate from Malawi”

**DOI:** 10.1128/MRA.00985-21

**Published:** 2021-10-21

**Authors:** Modupeh Betts, Seth Jarvis, Aaron Jeffries, Andrea Gori, Chrispin Chaguza, Jacquline Msefula, Caroline M. Weight, Brenda Kwambana-Adams, Neil French, Todd D. Swarthout, Jeremy S. Brown, Robert S. Heyderman

**Affiliations:** a NIHR Global Health Research Unit on Mucosal Pathogens, Division of Infection and Immunity, University College London, London, United Kingdom; b UCL Genetics Institute, University College London, London, United Kingdom; c University of Exeter, Exeter, United Kingdom; d Wellcome Sanger Institute, Wellcome Genome Campus, Hinxton, United Kingdom; e Darwin College, University of Cambridge, Cambridge, United Kingdom; f Department of Clinical Infection, Microbiology and Immunology, Institute of Infection, Veterinary and Ecological Sciences, University of Liverpool, Liverpool, United Kingdom; g Malawi-Liverpool-Wellcome Trust Clinical Research Programme Blantyre, Blantyre, Malawi; h Department of Respiratory Medicine, Centre for Inflammation and Tissue Repair, University College London, London, United Kingdom

## ERRATUM

Volume 10, no. 39, e00715-21, 2021, https://doi.org/10.1128/MRA.00715-21. Page 1, line 23: “>3 kb” should read “<3 kb.”

